# Determinants of Chronic Respiratory Symptoms among Pharmaceutical Factory Workers

**DOI:** 10.1155/2018/3815689

**Published:** 2018-01-31

**Authors:** Sahle Asfaw, Fikre Enquselassie, Yifokire Tefera, Muluken Gizaw, Samson Wakuma, Messay Woldemariam

**Affiliations:** ^1^Department of Preventive Medicine, School of Public Health, College of Health Sciences, Addis Ababa University, Addis Ababa, Ethiopia; ^2^Food, Beverage and Pharmaceutical Development Institute, Ministry of Industry, Addis Ababa, Ethiopia

## Abstract

**Background:**

Chronic respiratory symptoms including chronic cough, chronic phlegm, wheezing, shortness of breath, and chest pain are manifestations of respiratory problems which are mainly evolved as a result of occupational exposures. This study aims to assess determinants of chronic respiratory symptoms among pharmaceutical factory workers.

**Methods:**

A case control study was carried out among 453 pharmaceutical factory workers with 151 cases and 302 controls. Data was collected using pretested and structured questionnaire. The data was analyzed using descriptive statistics and bivariate and multivariate analysis.

**Result:**

Previous history of chronic respiratory diseases (AOR = 3.36, 95% CI = 1.85–6.12), family history of chronic respiratory diseases (AOR = 2.55, 95% CI = 1.51–4.32), previous dusty working environment (AOR = 2.26, 95% CI = 1.07–4.78), ever smoking (AOR = 3.66, 95% CI = 1.05–12.72), and service years (AOR = 1.86, 95% CI = 1.16–2.99) showed statistically significant association with chronic respiratory symptoms.

**Conclusion:**

Previous history of respiratory diseases, family history of chronic respiratory diseases, previous dusty working environment, smoking, and service years were determinants of chronic respiratory symptoms. Public health endeavors to prevent the burden of chronic respiratory symptoms among pharmaceutical factory workers should target the reduction of adverse workplace exposures and discouragement of smoking.

## 1. Introduction

“Chronic respiratory symptoms are defined as the development of one or more of the symptom(s) of chronic cough, chronic phlegm, chronic wheezing, chronic shortness of breath, and chronic chest tightness which last(s) at least three months in one year” [1]. At least one of these symptoms can be the manifestation of chronic respiratory diseases. Chronic respiratory diseases are a group of chronic diseases affecting the airways and the other structures of the lungs. Some of the most common ones are chronic obstructive pulmonary disease (including bronchitis and emphysema), asthma, pneumoconiosis, and chronic rhinosinusitis [2].

Globally, noncommunicable diseases (NCDs) were the leading cause of mortality which accounted for 38 million (68%) of the world's 56 million deaths in 2012. Four major NCDs (cardiovascular diseases, cancer, chronic respiratory diseases, and diabetes) were responsible for 82% of NCD deaths. Among those nontransmittable diseases, chronic respiratory diseases such as asthma and chronic obstructive pulmonary diseases represented 4 million or 10.7% deaths [3].

In 2011, International Labor Organization (ILO) revealed that occupational respiratory diseases represented up to 30% of all enrolled work related diseases and 10–20% of deaths were caused by respiratory conditions. Laborers in high hazard divisions, for example, mining, construction, and dust generating tasks have 50% prevalence of work related respiratory diseases [4]. In United Kingdom, there are around 12,000 deaths every year because of occupational respiratory diseases, of which around two-thirds were because of dust related diseases [5].

Workers employed in manufacturing, research, and development of pharmaceuticals are possibly exposed to drug substances in the working environment that are intended to change physiology and also to chemical precursors that are conceivably unsafe to health [6]. Allergies are relatively common in connection with exposure to a few compounds. Various instances of allergic rhinitis, asthma, and anaphylaxis have been experienced after inhaling seed powder of* Povata *seed, especially from powdered laxatives [7]. Penicillin and cephalosporin antibiotics and enzymes such as serratia peptidase and lysozyme chloride have been especially implicated to cause respiratory sensitization. Therapeutic chemical agents such as cimetidine, salbutamol, lisinopril, *α*-methyldopa, and opiates also cause respiratory sensitization [8].

In third world nations, where effective air contamination reduction strategies are inadequately available, individuals are constantly exposed to substances that can have negative health impacts in both the short and long term. Different risk factors have been related to chronic respiratory diseases, including sex, socioeconomic status, tobacco smoking habits, working environment, and polluting fuel utilized for private cooking/heating [9].

In Ethiopia, there is no systematic recording of occupational chronic respiratory disorders among workers in pharmaceutical factory. Respiratory disorders in these workers were high due to a number of different factors and great emphasis was not given before to control and prevent the problems due to lack of scientific information. Therefore, the aim of this study was to assess the determinants of chronic respiratory symptoms among pharmaceutical factory workers in Addis Ababa.

## 2. Methods

### 2.1. Study Design and Period

Institution based unmatched case control study design was conducted to assess determinants of chronic respiratory symptoms among pharmaceutical factory workers from February to April 2017.

### 2.2. Study Area

The study was conducted in four pharmaceutical factories in Addis Ababa Ethiopia. The factories manufacture a number of products of different therapeutic categories. There have been a total of 963 employees in the factories. From this, 730 employees were working in the production, quality control, research, and development departments.

### 2.3. Study Population

The study population was all employees of the selected pharmaceutical factories working in the production, quality control, research, and development departments with the following cases and controls definitions. Cases were workers who had chronic respiratory symptom(s) within one-year period in the pharmaceutical factory from February 2016 to February 2017. Controls were workers who had no chronic respiratory symptom(s) in the same period. A structured respiratory symptom questionnaire was used to identify cases and controls [10, 11]

Workers working in marketing department and other managerial working areas were excluded from the study assuming that they were less exposed to occupational chronic respiratory disease. Employees who were absent from work for more than 3 times of visit at the time of data collection were not included. Workers having work experience of below one year were excluded from the study.

### 2.4. Sample Size Determination

The sample size was calculated using EPI INFO version 7 statistical software StatCalc program for unmatched case control study design by considering 20% of workers exposed for greater than 5-year work experience in the factory among the control group from the previous study [1] with an assumption of one-to-two case-to-controls ratio, a minimum detectable odds ratio of 2 at 95% confidence interval, and 80% power of the study. Therefore, by considering the assumption, the sample size was determined. One hundred and thirty-seven cases and 274 controls were calculated and 10% none response rate was added for both cases and controls; finally a total of 453 study participants (151 cases and 302 controls) were included in the study.

### 2.5. Sampling Procedure

There are nine pharmaceutical manufacturing factories in Addis Ababa. From these, four factories were purposively selected because they had greater number of staff and have been producing relatively huge quantities of pharmaceuticals. Working departments which were included in the study were research and development, production, and quality control departments. Eligible study participants were identified by using workers' data obtained from human resource managers in each factory. The study samples that have been determined in the sample size determination were distributed in the four factories according to proportion number of workers. Prior to the actual data collection, a baseline survey was conducted for one week to identify source of cases and the control groups from each factory. Then a sampling frame was prepared for both cases and the control group for each factory based on survey data. The study subjects were selected by using simple random sampling technique (lottery method) from the sampling frame and by deriving an assumption that determinant factors of chronic respiratory symptoms were homogeneous in the factories.

The following operational definitions were used in the study.

- Chronic respiratory symptoms are defined as the development of one or more of the symptom(s) of chronic cough, chronic phlegm, chronic wheezing, chronic shortness of breath, and chronic chest tightness which last(s) at least three months in one year.
- Chronic cough is the development of cough as much as 4–6 times each day happening for most days of the week (≥four days) for at least three months in one year.
- Chronic phlegm is sputum expectoration as much as twice per day for most days of the week (≥4 days) for at least three months in one year.
- Chronic wheezing is a state of causing a wheezy or whistling sound during inspiration/expiration at least three months in a year occasionally apart from that caused by a cold or acute upper respiratory infection.
- Chronic chest tightness: chest pain with mucus that kept workers off work in the past year.
- Chronic shortness of breath: it is divided into 5 grades with the following definitions:
  1. Grade 0: no breathlessness except with strenuous exercise
  2. Grade 1: breathlessness when hurrying on the level ground or walking up a slight hill at least three months in a year
  3. Grade 2: walking slower than people of the same age on the same level because of breathlessness or need to stop for breath when walking at their own pace or level at least three months in a year
  4. Grade 3: stopping for breath after walking about a certain distance or a few minutes on the level ground at least three months in a year
  5. Grade 4: being too breathless to leave the house or breathless when dressing or undressing at least three months in a year
- Past dust exposure: any work experience on dusty environment before the current working position
- Past chemical/gas exposure: any work experience on chemical/gaseous environment before the current working position
- Family history of chronic respiratory diseases: the presence of one or more of the chronic diseases like chronic bronchitis, emphysema, tuberculosis (TB), heart disease, chronic sinus, asthma, and lung cancer in either of the natural parents (mother or father) identified by physicians
- Past chronic respiratory diseases: one or more of respiratory diseases like chronic bronchitis, emphysema, tuberculosis (TB), heart disease, chronic sinus, asthma, and lung cancer that could be developed before the current working position and identified by physicians
- Smoking behavior:
    Current smokers: workers who smoked at the time of the study or had stopped smoking less than one year before
    Ex-smokers: workers who had quit at least 1 year before the study
    Ever smoker: worker who has smoked at least one hundred cigarettes during the course of his/her life, which includes current smokers and ex-smokers

*Personal Protective Equipment (PPE). *Utilization of the worker-specialized clothing or equipment worn by employees helps to protect against health and safety hazards.

### 2.6. Measurement Tool

Pretested and structured questionnaire was used. The questionnaire used was a modified version of the American Thoracic Society and National Heart and Lung Institute, Division of Lung Disease Respiratory, questionnaire [12, 13]. It contains questions on sociodemographics, respiratory symptoms and behavioral, occupational, and environmental variables. The questionnaire was translated from English to Amharic and back to English.

### 2.7. Data Collection Procedure

Data was collected using pretested and structured Amharic version questionnaire via face to face interview of the study participants after getting ethical clearance from responsible bodies and informed verbal consent from study subjects. Based on the pretest, necessary modification was done on the questions and participants involved in the pretest were excluded in the actual data analysis. Data collection was administered by trained data collectors after two-day training.

### 2.8. Data Management and Quality Control

The questionnaire was prepared originally in English and translated to Amharic and back to English to keep the consistency. Training of the data collection team (data collectors and supervisors) on all aspects of data collection tools, questioning techniques, ethical issues, and role played on how to fill the questionnaire with pretesting in 5% of the sample before the actual study, was conducted for two days to ensure the quality of the data. Based on the pretest results, the questionnaire was additionally adjusted quantitatively, contextually, and terminologically and then administered to the study population. The data collected at the factory were checked daily for completeness, clarity, and logical consistency by the investigator and supervisors. Incorrectly filled or missed ones were sent back to the data collectors for correction. Anything which was unclear and ambiguous was corrected on the next day. Five percent of the samples were rechecked by the supervisor whether the interviewers have done their job correctly or not. Before the actual data processing, 5% of the collected data was entered to EPI INFO 7 software package twice by the principal investigator to verify proper entry and to maintain the quality of data.

### 2.9. Data Processing and Analysis

After the completion of data collection, the raw data was entered into a computer using EPI INFO 7 version computer software package for editing, cleaning, coding, and checking completeness and consistency. Finally, data was exported to Stata version 14 for data management and analysis. Descriptive statistics and bivariate logistic and multiple logistic regression analysis were done to see if there is an association between determinant factors and chronic respiratory symptoms. Crude odds ratio with 95% confidence intervals and significance level at *P* < 0.05 was used to assess the association between determinant factors and chronic respiratory symptoms. Variables with 95% confidence interval and *P* value at <0.05 during the bivariate analysis were included in the multiple logistic regression analysis to assess the relative effect of confounding variables. Adjusted odd ratios with 95% confidence interval were calculated.

### 2.10. Ethical Consideration

The study was approved by the Institutional Review Board (IRB) of Addis Ababa University, College of Health Sciences, School of Public Health Ethical Clearance Committee. Before data collection, a formal letter from Addis Ababa University School of Public Health Ethical Clearance Committee was submitted to the relevant and concerned bodies to the pharmaceutical factories. Verbal consent from each study subject was obtained after clear explanation on the purpose of the study. Confidentiality was maintained by omitting their names and personal identification.

## 3. Result

### 3.1. Sociodemographic Characteristics

A total of 453 participants with 151 cases and 302 controls were interviewed. From these, 83 (55.0%) of cases and 157 (52.0%) of controls were female workers; 102 (67.5%) of cases and 228 (75.5%) controls were below the age of 30 years. The religion frequency distribution showed that 109 (72.2%) of cases and 228 (75.5%) of controls were Orthodox. Relatively large number of participants in both cases, 94 (62.0%), and controls, 211 (69.9%), were single. Sixty-three (41.7%) of cases and 114 (37.7%) of controls had educational status of TVET and Diploma. Concerning employment condition, 121 (80.1%) of cases and 216 (71.5%) of controls were permanent employees. One hundred and one (66.9%) cases and two hundred and four (67.5%) controls earned below the mean monthly salary of the workers (2625 (±1980.75) Ethiopian birr) (Table 1).

### 3.2. Previous and Family History of Chronic Respiratory Diseases, Past Dust, and Chemicals/Gas Working Environment

From the study participants, 40 (26.5%) of cases and 25 (8.3%) of controls had reported the presence of chronic respiratory diseases identified by physicians before they started working in their current pharmaceutical factory job. Family history of chronic respiratory disease was reported by 45 (29.8%) cases and 37 (12.3%) controls. The study revealed that 60 (39.7%) of cases and 65 (21.5%) of controls had worked in dusty working environment before they started working their current job; 48 (31.8%) cases and 52 (17.2%) controls had been exposed to chemicals/gas working environment before they were employed in the pharmaceutical factory (Table 2).

### 3.3. Behavioral Characteristics

In this study, 11 (7.3%) of cases and 4 (1.3%) of controls were ever smokers (current and ex- smokers). From the total ever smokers, 2 (18.2%) cases and 2 (50.0%) controls were current smokers. Eighty-five (56.3%) cases and 159 (52.6%) controls reported that they drank alcohol. From alcohol drinker, 60 (70.6%) cases and 116 (73.0%) controls drank occasionally. One hundred and twenty-nine (85.4%) of cases and 263 (87.1%) of controls used personal respiratory protective material (Table 3).

### 3.4. Occupational and Environmental Factors

From the respondents, 121 (80.1%) of cases and 214 (70.8%) of controls were working in the production department of the pharmaceutical factories. Eighty (53.0%) cases and 171 (56.6%) controls worked for more than 48 hours per week. Eighty-five (56.3%) of cases and 77 (25.5%) controls had work experience of greater than five years. One hundred and eight (71.5%) cases and 204 (67.5%) controls used electricity as a source of energy in their home (Table 4).

### 3.5. Factors Associated with Chronic Respiratory Symptoms: Bivariate Analysis

Sex, age, religion, marital status, educational level, and monthly salary of the workers did not show significant association with chronic respiratory symptoms in the bivariate analysis. Employment condition of the workers had significant association with chronic respiratory symptoms. Permanently employed workers (Crude Odd Ratio (COR = 1.61, 95% CI = (1.01–2.57)) were more likely to have chronic respiratory symptoms than temporary workers.

Workers who had previous history of chronic respiratory diseases (COR = 3.99, 95% Cl = (2.31–6.89)) were more likely to have chronic respiratory symptom(s) than those who had no history. The odds of developing chronic respiratory symptom(s) were significantly higher for those workers who had family history of chronic respiratory diseases (COR = 3.04, 95% Cl = (1.86–4.96)) than those who had not.

Workers of the pharmaceutical factory who had previous dust exposure (COR = 2.40, 95% Cl = (1.57–3.68)) were more likely to have chronic respiratory symptom(s) than those workers who did not work in dusty environment. Past chemicals/gaseous exposure was also significantly associated with chronic respiratory symptoms. Workers who had worked in previous chemicals/gaseous working environment had the odds of developing chronic respiratory symptoms about 2 times more likely (COR = 2.24, 95% CI = (1.42–3.53) than those who did not.

The odds of developing chronic respiratory symptom(s) were significantly higher for those workers who smoked cigarettes at any time in their life (COR = 5.85, 95% Cl = (1.83–18.70)) than nonsmokers. However, current smoking, alcohol drinking, khat chewing, and use of personal respiratory protective material did not show significant association with chronic respiratory symptoms among pharmaceutical factory workers in Addis Ababa.

Among environmental and occupational factors, working department, total working hours per week, training on respiratory health, and energy used at home did not show significant association with chronic respiratory symptoms. However, work experience (service years) showed association with development of chronic respiratory symptoms. Workers of the pharmaceutical factory who had service years greater than five years had the odds of developing chronic respiratory symptoms about two times more likely (COR = 2.27, 95% CI = (1.50–3.43)) than those workers with work experience of one to five years (Table 5).

### 3.6. Multiple Variable Analysis

The multivariate analysis indicated that previous history of chronic respiratory diseases, family history of chronic respiratory diseases, previous dusty working environment, ever smoking, and service years showed statistically significant association with chronic respiratory symptoms among pharmaceutical factory workers. But, employment condition and past chemical/gas working environment did not show significant association with chronic respiratory symptoms (Table 6).

## 4. Discussion

This study identified determinants of chronic respiratory symptoms among pharmaceutical factory workers in Addis Ababa. Previous history of chronic respiratory diseases, family history of chronic respiratory diseases, previous dusty working environment, ever smoking, and service years were the independent determinants of chronic respiratory symptoms of the workers. But, it was found that none of the sociodemographic characteristics were statistically significant with the development of chronic respiratory symptoms.

The study showed that workers who had previous history of respiratory diseases were about 3 times more likely (AOR = 3.36, 95% CI = (1.85–6.12)) to develop chronic respiratory symptoms than those who had not the history of the disease. This finding was consistent with the study done in Ethiopia [1]. This can be explained by the fact that the previous respiratory disease might affect the normal function of the respiratory system by causing airway obstruction and respiratory sensitization. This alteration may lead to the development of chronic respiratory symptoms. Preexisting respiratory diseases may impair respiratory tract defense mechanism causing the increased susceptibility to the occurrence the symptoms. Asthmatics have a unique kind of inflammation in the airways. This makes them more responsive than nonasthmatics to a wide range of triggers, leading to excessive narrowing with consequent reduced airflow and symptomatic wheezing and dyspnea.

This study indicated that employees of the factory in which either of their natural parents (mother or father) had history of chronic respiratory diseases were associated with chronic respiratory symptoms. Workers who had family history of chronic respiratory diseases were about 3 times more likely to develop chronic respiratory symptoms than those who had no disease. This finding was in line with a study done in Thailand [10]. This may be due to the fact that genetics has its own contribution for the development of chronic respiratory symptoms.

Employees of the pharmaceutical factory, who had worked in any dusty working environment before their current job, were about 2 times more likely (AOR = 2.26, 95% CI = ((1.07–4.78)) to develop chronic respiratory symptoms than workers that were not engaged in previous dusty work environment. This result is in line with the study done in United States and China [14, 15]. This statistical significance could be because the workers might have previously worked in dusty jobs identified to cause respiratory problems including organic dust [16], cement dust exposure [17], and aerosol and sisal fiber dust [18]. Exposures to inorganic and organic dust may lead to interstitial lung disease that presents with a restrictive pattern and a decreased diffusing capacity. Similarly, exposures to many chemical agents may result in occupational asthma or COPD which is characterized by airway obstruction. The past exposure to dust might also lead to the aforementioned respiratory tissue physiologic change in a later life and exacerbate the occurrence of respiratory symptoms.

Ever smoker workers (ex and current smokers) were about 4 times more likely to have chronic respiratory symptoms than nonsmokers. This finding was consistent with the studies done in South Africa, Croatia, and India [9, 13, 15, 19, 20]; however, it was inconsistent with a study done in Iran, which reported no difference in respiratory symptoms between ever smokers and nonsmokers [21]. This disparity in result might be due to the difference in the frequency and duration of smoking, strength, quality, and number of cigarettes smoked.

Workers who had service greater than five years had the odds of developing chronic respiratory symptoms about two times more likely than those workers with work experience of one to five years. The result was in line with study done by many researchers [15, 19, 22–24]. This might be due to increased dust accumulation in the respiratory system with long-term exposure leading to air way limitations. The finding indicated that long duration of exposures to the manufacturing of pharmaceuticals may lead to the development of chronic respiratory symptoms. Many authors reported that rhinitis, occupational asthma, and symptoms in pharmaceutical workers may be due to exposures to groups of antibiotics such as penicillin, cephalosporin, tetracycline, azithromycin, spiramycin, and other therapeutic agents including salbutamol, cimetidine, hydroxychloroquine, lisinopril, *α*-methyldopa, hydralazine, and opiates [7, 8, 19, 25]. In the study settings, at least one of the aforementioned medicines is manufactured in each factory which may contribute to the development of long lasting respiratory symptoms and diseases.

The study revealed that sociodemographic characteristics such as sex, age, religion, marital status, educational status, employment condition, and monthly income of workers were not significantly associated with the development of chronic respiratory symptoms. Previous study has reported that sex and socioeconomic status had significant association with chronic respiratory diseases [9, 19]. This difference may account for the existence of variation among countries' culture, belief, and living and earning conditions.

The finding of the study indicated that there was no difference in the development of chronic respiratory symptoms between those workers who used personal respiratory protective equipment and those that did not in pharmaceutical factory. This finding was in line with the study done in Ethiopia and Tanzania [1, 26]. This might be due to the users that could not use appropriate PPE in terms of quality (piece of cloth instead of the nose/mouth mask or respirator), protection capacity, and comfort.

The finding of this study had some strength. This case control study design easily identifies multiple exposures for chronic respiratory symptoms by comparing the exposures among workers who experienced chronic respiratory symptoms and who did not. Use of face to face interview during data collection reduced nonresponse rate, permitted clarification of questionnaires, and addressed all participants who differ in sociodemographic status. The study has also limitations. A one-year case control study design could lead to recall bias (under- or overreporting of some determinant factors of chronic respiratory symptoms).

The study showed that occupational exposure time (service years of workers) in pharmaceutical factory was determinant of chronic respiratory symptoms of the workers. In addition to occupational exposure time, factors such as previous history of chronic respiratory diseases, family history of chronic respiratory diseases, previous dusty working environment, and ever smoking were determinants of chronic respiratory symptoms among pharmaceutical factory workers in Addis Ababa.

Comprehensive occupational safety practice on occupational chronic respiratory disorders should be done by considering the determinant factors in the factories. Workers of the pharmaceutical factories with previous history of chronic respiratory diseases, family history of chronic respiratory diseases, and previous dusty working environment should recognize stimulating agents and take action accordingly. Smoking discouragement should also be promoted. Long serving workers with complicated chronic respiratory symptoms should get treatment. Further prospective study should be done to identify causes of chronic respiratory symptoms in the pharmaceutical factories.

## Figures and Tables

**Table 1:** Distribution of sociodemographic characteristics of participants in pharmaceutical factories in Addis Ababa, Ethiopia, May 2017.

Socio demographic variables	Case (*n* = 151) (%)	Control (*n* = 302) (%)	Total (%)	*P* value
Sex				
Female	83 (55.0)	157 (52.0)	240 (53)	0.549
Male	68 (45.0)	145 (48.0)	213 (47)	
Age group				
<30 years	102 (67.5)	228 (75.5)	330 (72.8)	
≥30 years	49 (32.5)	74 (24.5)	123 (27.2)	0.074
Religion				
Orthodox	109 (72.2)	228 (75.5)	337 (74.4)	0.114
Catholic	3 (2.0)	1 (0.3)	4 (0.9)	
Muslim	15 (9.9)	28 (9.3)	43 (9.5)	0.150
Protestant	24 (15.9)	45 (14.9)	69 (15.2)	0.144
Marital status				
Married	55 (36.0)	86 (28.5)	141 (31.1)	0.842
Single	94 (62.0)	211 (69.9)	305 (67.3)	0.925
Widowed	1 (1.0)	2 (1.0)	3 (0.7)	
Divorced	1 (1.0)	3 (10.0)	4 (0.9)	0.810
Educational level				
≤ grade 8	2 (1.3)	6 (2.0)	8 (1.8)	
Grades 9–12	59 (39.1)	109 (36.1)	168 (37.1)	0.560
Technical and vocational education training (TVET)	63 (41.7)	114 (37.7)	177 (39.1)	0.543
First degree & above	27 (17.9)	73 (24.2)	100 (22.1)	0.902
Employment condition				
Temporary	30 (19.9)	86 (28.5)	116 (25.6)	
Permanent	121 (80.1)	216 (71.5)	337 (74.4)	0.049
Monthly salary in ETB				
≤2625	101 (66.9)	204 (67.5)	305 (67.3)	
>2625	50 (33.1)	98 (32.5)	148 (32.7)	0.887

**Table 2:** Distribution of previous and family history of CRDs and past dust and chemicals/gas working environment of participants in pharmaceutical factories in Addis Ababa, Ethiopia, May 2017.

Variables	Case (*n* = 151) (%)	Control (*n* = 302) (%)	Total (%)	*P* value
Previous history of chronic respiratory diseases				
Yes	40 (26.5)	25 (8.3)	65 (14.3)	<0.0001
No	111 (73.5)	277 (91.7)	388 (85.7)	
Family history of chronic respiratory disease				
Yes	45 (29.8)	37 (12.3)	82 (18.1)	<0.0001
No	106 (70.2)	265 (87.7)	371 (81.9)	
Previous dusty working environment				
Yes	60 (39.7)	65 (21.5)	125 (27.6)	<0.0001
No	91 (60.3)	237 (78.5)	328 (72.4)	
Previous chemicals/gas working environment				
Yes	48 (31.8)	52 (17.2)	100 (22.1)	0.001
No	103 (68.2)	250 (82.8)	353 (77.9)	

**Table 3:** Distribution of behavioral factors of participants in pharmaceutical factories in Addis Ababa, Ethiopia, May 2017.

Behavioral factors	Case (*n* = 151) (%)	Control (*n* = 302) (%)	Total (%)	*P* value
Ever smokers (current and ex-smokers)				
Yes	11 (7.3)	4 (1.3)	15 (3.3)	0.003
No	140 (92.7)	298 (98.7)	438 (96.7)	
Current smokers				
Yes	2 (18.2)	2 (50)	4 (26.7)	0.236
No	9 (81.8)	2 (50)	11 (73.3)	
Alcohol drinking				
Yes	85 (56.3)	159 (52.6)	244 (53.9)	0.73
No	66 (43.7)	143 (47.4)	209 (46.1)	
How often?				
Everyday	1 (1.2)	2 (1.2)	3 (1.2)	
One–three days/week	24 (28.2)	41 (25.8)	65 (26.7)	0.900
Occasionally	60 (70.6)	116 (73.0)	176 (72.1)	0.978
Khat chewing				
Yes	19 (12.6)	25 (8.3)	44 (9.7)	0.147
No	132 (87.4)	277 (91.7)	409 (90.3)	
How often?				
Everyday	1 (5.3)	1 (4.0)	2 (4.5)	
One–three days/week	6 (31.6)	6 (24.0)	12 (27.3)	1.000
Occasionally	12 (63.1)	18 (72.0)	30 (68.2)	0.782
Use of personal respiratory protective material				
Yes	129 (85.4)	263 (87.1)	392 (86.5)	
No	22 (14.6)	39 (12.9)	61 (13.5)	0.627

**Table 4:** Distribution of occupational and environmental factors of participants in pharmaceutical factories in Addis Ababa, Ethiopia, May 2017.

Occupational and environmental factors	Case (*n* = 151) (%)	Control (*n* = 302) (%)	Total (%)	*P* value
Working department				
Research and development	16 (10.6)	44 (14.6)	60 (13.2)	0.753
Production	121 (80.1)	214 (70.8)	335 (74.0)	0.079
Quality control	14 (9.3)	44 (14.6)	58 (12.8)	
Working hours per week				
≤48 hrs	71 (47.0)	131 (43.4)	202 (44.6)	
>48 hrs	80 (53.0)	171 (56.6)	251 (55.4)	0.462
Length of time worked				
1–5 years	66 (43.7)	225 (74.5)	310 (68.4)	
>5 years	85 (56.3)	77 (25.5)	143 (31.6)	<0.0001
Training on respiratory health				
Yes	52 (34.4)	100 (33.1)	152 (33.6)	0.778
No	99 (65.6)	202 (66.9)	301 (66.4)	
Energy used at home				
Electricity	108 (71.5)	204 (67.5)	312 (68.9)	0.377
kerosene	16 (10.6)	28 (9.3)	44 (9.7)	0.357
Wood	1 (0.7)	5 (1.7)	6 (1.3)	
Charcoal	26 (17.2)	65 (21.5)	91 (20.1)	0.536

**Table 5:** Bivariate analysis of environmental and occupational factors and chronic respiratory symptoms among pharmaceutical factory workers in Addis Ababa, Ethiopia, May 2017.

Occupational and environmental factors	Case (*n* = 151) (%)	Control (*n* = 302) (%)	COR (95% CI)	*P* value
Working department				
Research and development	16 (10.6)	44 (14.6)	1.14 (0.50–2.62)	0.753
Production	121 (80.1)	214 (70.8)	1.78 (0.94–3.38)	0.079
Quality control	14 (9.3)	44 (14.6)	1	
Working hours per week				
≤48 hrs	71 (47.0)	131 (43.4)	1	
>48 hrs	80 (53.0)	171 (56.6)	0.86 (0.58–1.28)	0.462
Length of time worked				
1–5 years	66 (43.7)	225 (74.5)	1	
>5 years	85 (56.3)	77 (25.5)	2.27 (1.50–3.43)	<0.0001
Training on respiratory health				
Yes	52 (34.4)	100 (33.1)	1.06 (0.7–1.6)	0.778
No	99 (65.6)	202 (66.9)	1	
Energy used at home				
Electricity	108 (71.5)	204 (67.5)	2.65 (0.31–22.95)	0.377
kerosene	16 (10.6)	28 (9.3)	2.86 (0.31–26.66)	0.357
Wood	1 (0.7)	5 (1.7)	1	
Charcoal	26 (17.2)	65 (21.5)	1.99 (0.22–17.95)	0.536

**Table 6:** Multiple logistic regression model of associated factors and chronic respiratory symptoms among pharmaceutical factory workers in Addis Ababa, Ethiopia, May 2017.

Variables	Case (*n* = 151) (%)	Control (*n* = 302) (%)	COR (95% CI)	Adjusted odd ratio (AOR) (95% CI)
Employment condition				
Temporary	30 (19.9)	86 (28.5)	1	1
Permanent	121 (80.1)	216 (71.5)	1.61 (1.01–2.57)	0.98 (0.57–1.66)
Previous history of respiratory diseases				
Yes	40 (26.5)	25 (8.3)	3.99 (2.31–6.89)	3.36 (1.85–6.12)^*∗∗*^
No	111 (73.5)	277 (91.7)	1	1
Family history of chronic respiratory disease				
Yes	45 (29.8)	37 (12.3)	3.04 (1.86–4.96)	2.55 (1.51–4.32)^*∗∗*^
No	106 (70.2)	265 (87.7)	1	1
Previous dusty working environment				
Yes	60 (39.7)	65 (21.5)	2.40 (1.57–3.68)	2.26 (1.07–4.78)^*∗*^
No	91 (60.3)	237 (78.5)	1	1
Previous chemicals/gas working environment				
Yes	48 (31.8)	52 (17.2)	2.24 (1.42–3.53)	1.10 (0.49–2.48)
No	103 (68.2)	250 (82.8)	1	1
Ever smokers (current and ex-smokers)				
Yes	11 (7.3)	4 (1.3)	5.85 (1.83–18.70)	3.66 (1.05–12.72)^*∗*^
No	140 (92.7)	298 (98.7)	1	1
Length of time worked				
1–5 years	66 (43.7)	225 (74.5)	1	1
>5 years	85 (56.3)	77 (25.5)	2.27 (1.50–3.43)	1.86 (1.16–2.99)^*∗*^

^*∗*^Significant at *P* < 0.05; ^*∗∗*^significant at *P* < 0.001.
